# 2-Isopropyl-5-methyl­cyclo­hexyl quinoline-2-carboxyl­ate

**DOI:** 10.1107/S1600536813033060

**Published:** 2013-12-11

**Authors:** E. Fazal, Jerry P. Jasinski, Brian J. Anderson, B. S. Sudha, S. Nagarajan

**Affiliations:** a Department of Chemistry, Yuvaraja’s College, Mysore 570 005, India; b Department of Chemistry, Keene State College, 229 Main Street, Keene, NH 03435-2001, USA; c P.P.S.F.T. Department, Central Food Technplogy Research institute, Mysore 570 005, India

## Abstract

In the title compound, C_20_H_25_NO_2_, the cyclo­hexyl ring adopts a slightly disordered chair conformation. The dihedral angle between the mean planes of the quinoline ring and the carboxyl­ate group is 22.2 (6)°. In the crystal, weak C—H⋯N inter­actions make chains along [010].

## Related literature   

For heterocycles in natural products, see: Morimoto *et al.* (1991 ▶); Michael (1997 ▶). For heterocycles in fragrances and dyes, see: Padwa *et al.* (1999 ▶). For heterocycles in biologically active compounds, see: Markees *et al.* (1970 ▶); Campbell *et al.*(1988 ▶). For quinoline alkaloids used as efficient drugs for the treatment of malaria, see: Robert & Meunier, (1998 ▶). For quinoline as a privileged scaffold in cancer drug discovery, see: Solomon & Lee (2011 ▶). For related structures, see: Fazal *et al.* (2012 ▶, 2013*a* ▶,*b* ▶,*c* ▶); Butcher *et al.* (2007 ▶); Jing & Qin (2008 ▶); Jasinski *et al.* (2010 ▶). For puckering parameters, see Cremer & Pople (1975 ▶).
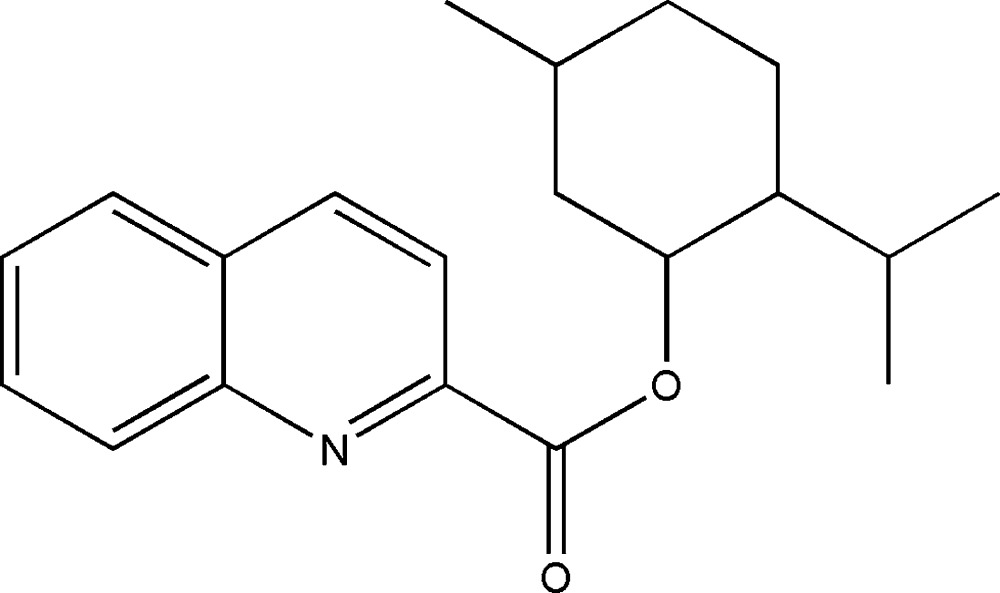


## Experimental   

### 

#### Crystal data  

C_20_H_25_NO_2_
*M* *_r_* = 311.41
Orthorhombic, 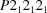

*a* = 9.31412 (17) Å
*b* = 11.9669 (2) Å
*c* = 15.4894 (3) Å
*V* = 1726.47 (6) Å^3^
*Z* = 4
Cu *K*α radiation
μ = 0.60 mm^−1^
*T* = 173 K
0.38 × 0.32 × 0.24 mm

#### Data collection  

Agilent Gemini EOS diffractometer
Absorption correction: multi-scan (*CrysAlis PRO* and *CrysAlis RED*; Agilent, 2012 ▶). *T*_min_ = 0.921, *T*_max_ = 1.000
11010 measured reflections
3389 independent reflections
3281 reflections with *I* > 2σ(*I*)
*R*_int_ = 0.037

#### Refinement  

*R*[*F* ^2^ > 2σ(*F* ^2^)] = 0.037
*wR*(*F* ^2^) = 0.098
*S* = 1.04
3389 reflections
212 parameters
H-atom parameters constrained
Δρ_max_ = 0.20 e Å^−3^
Δρ_min_ = −0.17 e Å^−3^
Absolute structure: Flack (1983 ▶); 1372 Friedel pairs
Absolute structure parameter: −0.01 (13)

### 

Data collection: *CrysAlis PRO* (Agilent, 2012 ▶); cell refinement: *CrysAlis PRO*; data reduction: *CrysAlis RED* (Agilent, 2012 ▶); program(s) used to solve structure: *SUPERFLIP* (Palatinus & Chapuis, 2007 ▶); program(s) used to refine structure: *SHELXL2012* (Sheldrick, 2008 ▶); molecular graphics: *OLEX2* (Dolomanov *et al.*, 2009 ▶); software used to prepare material for publication: *OLEX2*.

## Supplementary Material

Crystal structure: contains datablock(s) I. DOI: 10.1107/S1600536813033060/tk5278sup1.cif

Structure factors: contains datablock(s) I. DOI: 10.1107/S1600536813033060/tk5278Isup2.hkl

Supporting information file. DOI: 10.1107/S1600536813033060/tk5278Isup3.cml

Additional supporting information:  crystallographic information; 3D view; checkCIF report

## Figures and Tables

**Table 1:** Hydrogen-bond geometry (Å, °)

*D*—H⋯*A*	*D*—H	H⋯*A*	*D*⋯*A*	*D*—H⋯*A*
C7—H7⋯N1^i^	0.95	2.56	3.509 (2)	174

Symmetry code: (i) 
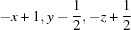
.

**Fig. 1.**
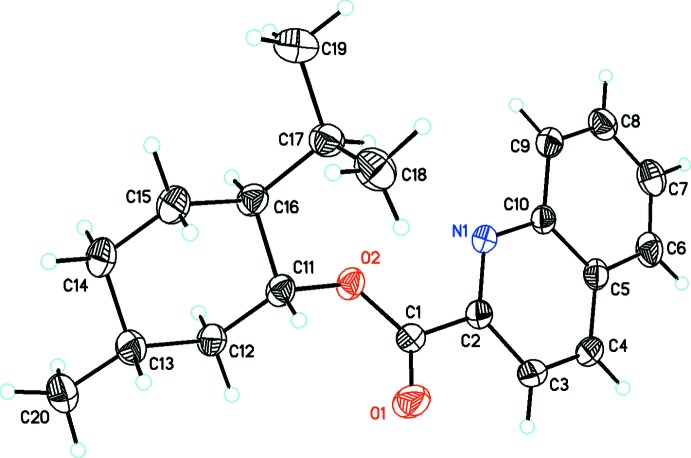
ORTEP drawing of (I) showing the labeling scheme with 50% probability displacement ellipsoids.

**Fig. 2.**
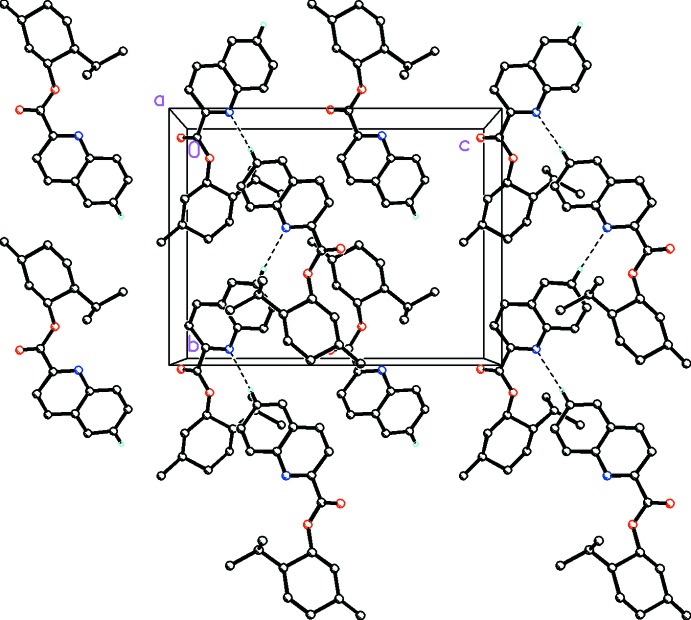
Molecular packing for (I) viewed along the a axis. Dashed lines indicate weak C7—H7···N1 intermolecular interactions making chains along [0 1 0]. The remaining H atoms have been removed for clarity.

## supplementary crystallographic information

### 1. Comment

Quinoline-2 carboxylic acid derivatives are a class of important materials as anti-tuberculosis agents, as fluorescent reagents, hydrophobic field-detection reagents, visualisation reagents, fluorescent labelled peptide probes and as antihyperglycemics. Quinoline derivatives represent a major class of heterocycles and are found in natural products (Morimoto *et al.*, 1991; Michael, 1997), numerous commercial products, including fragrances, dyes (Padwa *et al.*, 1999) and biologically active compounds (Markees *et al.*, 1970; Campbell *et al.*, 1988). Quinoline alkaloids such as quinine, chloroquin, mefloquine and amodiaquine are used as efficient drugs for the treatment of malaria (Robert & Meunier, 1998). Quinoline as a privileged scaffold in cancer drug discovery is published (Solomon & Lee, 2011). The crystal structures of 4-methylphenyl quinoline-2-carboxylate (Fazal *et al.*, 2012), 4-chloro-3-methylphenyl quinoline-2- carboxylate (Fazal *et al.*, 2013*a*), 4-chlorophenyl quinoline- 2-carboxylate (Fazal *et al.*, 2013*b*), 3,4-dimethylphenyl quinoline-2-carboxylate (Fazal *et al.*, 2013*c*), 1-(quinolin-2-yl)ethanone (Butcher *et al.*, 2007) and methyl quinoline-2-carboxylate (Jing & Qin, 2008) as well as the synthesis, crystal structures and theoretical studies of four Schiff bases derived from 4-hydrazinyl-8-(trifluoromethyl) quinoline (Jasinski *et al.*, 2010) have been reported. In view of the importance of quinolines, this paper reports the crystal structure of the title compound, (I), C_20_H_25_NO_2_.

In the title compound, (I), Fig. 1, the cyclohexyl ring adopts a slightly disordered chair conformation (puckering parameters for C11–C16: Q, θ, and φ = 0.593 (2)Å, 4.32 (19)° and 308 (2)°, respectively (Cremer & Pople, 1975). The dihedral angle between the mean planes of the quinoline ring and the carboxylate group (C2/C1/O1/O2) is 22.2 (6)°. In the crystal, weak C7—H7···N1 intermolecular interactions make chains along [0 1 0] and influence the crystal packing (Fig. 2 & Table 1).

### 2. Experimental

The title compound was prepared by the following procedure: To a mixture of 1.73 g (10 mmol) of quinaldic acid and 1.56 g (10 mmole) of 2-isopropyl-5-methylcyclohexanol in a round-bottomed flask fitted with a reflux condenser with a drying tube is added phosphorous oxychloride (0.150 g, 10 mmol). The mixture is heated with occasional swirling, and temperature is maintained at 348-353 K. At the end of 8 h the reaction mixture is poured in to a solution of sodium bicarbonate (2 g) in water (25 mL). The precipitated ester is collected on a filter and washed with water. The yield of crude, air dried 2-isopropyl-5-methylcyclohexyl quinoline-2-carboxylate is isolated in 1.71 to 1.85 g (65-70 %) yield. X-ray quality crystals were obtained by recrystallization from absolute ethanol by slow evaporation (M.pt: 414-416 K).

### 3. Refinement

All of the H atoms were placed in their calculated positions and then refined using the riding model with C—H lengths of 0.95–1.00 Å, and with *U*_iso_(H) = 1.2–1.5*U*_eq_(C). Two reflections, *i.e*. (1 0 1) and (0 0 2), were removed from the final cycles of refinement owing to poor agreement.

### Crystal data

**Table:**

C_20_H_25_NO_2_	*D*_x_ = 1.198 Mg m^−^^3^
*M**_r_* = 311.41	Cu *K*α radiation, λ = 1.54184 Å
Orthorhombic, *P*2_1_2_1_2_1_	Cell parameters from 6294 reflections
*a* = 9.31412 (17) Å	θ = 4.7–72.3°
*b* = 11.9669 (2) Å	µ = 0.60 mm^−^^1^
*c* = 15.4894 (3) Å	*T* = 173 K
*V* = 1726.47 (6) Å^3^	Irregular, colourless
*Z* = 4	0.38 × 0.32 × 0.24 mm
*F*(000) = 672	

### Data collection

**Table:**

Agilent Gemini EOS diffractometer	3389 independent reflections
Radiation source: Enhance (Cu) X-ray Source	3281 reflections with *I* > 2σ(*I*)
Detector resolution: 16.0416 pixels mm^-1^	*R*_int_ = 0.037
ω scans	θ_max_ = 72.4°, θ_min_ = 4.7°
Absorption correction: multi-scan (*CrysAlis PRO* and *CrysAlis RED*; Agilent, 2012).	*h* = −11→5
*T*_min_ = 0.921, *T*_max_ = 1.000	*k* = −14→14
11010 measured reflections	*l* = −19→18

### Refinement

**Table:**

Refinement on *F*^2^	H-atom parameters constrained
Least-squares matrix: full	*w* = 1/[σ^2^(*F*_o_^2^) + (0.0661*P*)^2^ + 0.1484*P*] where *P* = (*F*_o_^2^ + 2*F*_c_^2^)/3
*R*[*F*^2^ > 2σ(*F*^2^)] = 0.037	(Δ/σ)_max_ < 0.001
*wR*(*F*^2^) = 0.098	Δρ_max_ = 0.20 e Å^−^^3^
*S* = 1.04	Δρ_min_ = −0.17 e Å^−^^3^
3389 reflections	Extinction correction: *SHELXL2012* (Sheldrick, 2008), Fc^*^=kFc[1+0.001xFc^2^λ^3^/sin(2θ)]^-1/4^
212 parameters	Extinction coefficient: 0.0093 (10)
0 restraints	Absolute structure: Flack (1983); 1372 Friedel pairs
Primary atom site location: structure-invariant direct methods	Absolute structure parameter: −0.01 (13)
Hydrogen site location: inferred from neighbouring sites	

### Special details

**Table:**

Geometry. All esds (except the esd in the dihedral angle between two l.s. planes) are estimated using the full covariance matrix. The cell esds are taken into account individually in the estimation of esds in distances, angles and torsion angles; correlations between esds in cell parameters are only used when they are defined by crystal symmetry. An approximate (isotropic) treatment of cell esds is used for estimating esds involving l.s. planes.

### Fractional atomic coordinates and isotropic or equivalent isotropic displacement parameters (Å^2^)

**Table:**

	*x*	*y*	*z*	*U*_iso_*/*U*_eq_	
O1	0.92164 (17)	0.54532 (12)	0.51535 (10)	0.0408 (4)	
O2	0.79892 (13)	0.64179 (10)	0.41465 (8)	0.0261 (3)	
N1	0.70130 (16)	0.45417 (11)	0.34655 (9)	0.0230 (3)	
C1	0.83460 (19)	0.55000 (15)	0.45755 (11)	0.0253 (4)	
C2	0.75365 (19)	0.44942 (14)	0.42566 (11)	0.0240 (4)	
C3	0.7426 (2)	0.35518 (16)	0.48000 (11)	0.0295 (4)	
H3	0.7848	0.3554	0.5358	0.035*	
C4	0.6697 (2)	0.26358 (15)	0.45059 (12)	0.0311 (4)	
H4	0.6592	0.1997	0.4864	0.037*	
C5	0.6104 (2)	0.26440 (14)	0.36683 (12)	0.0262 (4)	
C6	0.5318 (2)	0.17400 (15)	0.33109 (14)	0.0327 (4)	
H6	0.5171	0.1083	0.3643	0.039*	
C7	0.4772 (2)	0.18048 (17)	0.24958 (15)	0.0348 (4)	
H7	0.4233	0.1199	0.2268	0.042*	
C8	0.5004 (2)	0.27692 (16)	0.19885 (13)	0.0314 (4)	
H8	0.4633	0.2800	0.1418	0.038*	
C9	0.5755 (2)	0.36578 (15)	0.23068 (12)	0.0283 (4)	
H9	0.5908	0.4299	0.1957	0.034*	
C10	0.63087 (19)	0.36241 (14)	0.31610 (11)	0.0235 (4)	
C11	0.88592 (18)	0.74196 (14)	0.42860 (11)	0.0243 (4)	
H11	0.9840	0.7195	0.4483	0.029*	
C12	0.8164 (2)	0.81503 (14)	0.49708 (12)	0.0265 (4)	
H12A	0.8105	0.7732	0.5521	0.032*	
H12B	0.7174	0.8342	0.4790	0.032*	
C13	0.9028 (2)	0.92258 (15)	0.51090 (12)	0.0290 (4)	
H13	0.9993	0.9015	0.5340	0.035*	
C14	0.9247 (2)	0.98221 (15)	0.42454 (14)	0.0345 (5)	
H14A	0.8310	1.0098	0.4033	0.041*	
H14B	0.9880	1.0477	0.4334	0.041*	
C15	0.9910 (2)	0.90594 (16)	0.35660 (13)	0.0329 (4)	
H15A	1.0879	0.8826	0.3756	0.039*	
H15B	1.0012	0.9474	0.3016	0.039*	
C16	0.89728 (19)	0.80190 (15)	0.34186 (12)	0.0259 (4)	
H16	0.7988	0.8290	0.3269	0.031*	
C17	0.9465 (2)	0.72518 (16)	0.26757 (12)	0.0301 (4)	
H17	0.8757	0.6626	0.2637	0.036*	
C18	1.0935 (2)	0.67304 (19)	0.28311 (15)	0.0397 (5)	
H18A	1.1647	0.7323	0.2919	0.060*	
H18B	1.1208	0.6280	0.2329	0.060*	
H18C	1.0896	0.6253	0.3345	0.060*	
C19	0.9430 (3)	0.7870 (2)	0.18104 (14)	0.0469 (6)	
H19A	0.8472	0.8188	0.1719	0.070*	
H19B	0.9652	0.7347	0.1343	0.070*	
H19C	1.0143	0.8472	0.1816	0.070*	
C20	0.8303 (2)	0.99877 (17)	0.57671 (14)	0.0370 (5)	
H20A	0.7301	1.0109	0.5600	0.056*	
H20B	0.8806	1.0706	0.5786	0.056*	
H20C	0.8337	0.9637	0.6338	0.056*	

### Atomic displacement parameters (Å^2^)

**Table:**

	*U*^11^	*U*^22^	*U*^33^	*U*^12^	*U*^13^	*U*^23^
O1	0.0551 (9)	0.0303 (7)	0.0368 (8)	−0.0097 (7)	−0.0205 (7)	0.0059 (6)
O2	0.0273 (6)	0.0196 (6)	0.0312 (6)	−0.0035 (5)	−0.0044 (5)	0.0018 (5)
N1	0.0272 (7)	0.0183 (6)	0.0235 (7)	0.0013 (6)	−0.0003 (6)	0.0007 (5)
C1	0.0306 (8)	0.0225 (8)	0.0228 (8)	−0.0003 (7)	0.0004 (7)	0.0008 (7)
C2	0.0267 (8)	0.0217 (8)	0.0237 (8)	0.0019 (7)	0.0013 (7)	−0.0004 (7)
C3	0.0405 (10)	0.0248 (8)	0.0232 (8)	−0.0003 (8)	−0.0017 (7)	0.0021 (7)
C4	0.0448 (10)	0.0211 (8)	0.0273 (9)	−0.0001 (7)	0.0038 (8)	0.0053 (7)
C5	0.0297 (8)	0.0194 (8)	0.0294 (9)	0.0005 (7)	0.0053 (7)	−0.0012 (7)
C6	0.0376 (10)	0.0214 (8)	0.0393 (11)	−0.0050 (7)	0.0048 (8)	−0.0029 (7)
C7	0.0328 (10)	0.0288 (9)	0.0428 (11)	−0.0054 (8)	−0.0005 (9)	−0.0104 (8)
C8	0.0302 (9)	0.0325 (9)	0.0316 (9)	0.0036 (8)	−0.0051 (8)	−0.0078 (8)
C9	0.0306 (9)	0.0252 (9)	0.0291 (9)	0.0040 (7)	−0.0020 (7)	−0.0011 (7)
C10	0.0255 (8)	0.0198 (8)	0.0252 (8)	0.0031 (7)	0.0020 (6)	−0.0012 (6)
C11	0.0242 (7)	0.0205 (8)	0.0281 (8)	−0.0043 (7)	−0.0026 (6)	0.0017 (7)
C12	0.0289 (9)	0.0229 (8)	0.0279 (8)	−0.0052 (7)	−0.0006 (7)	0.0008 (7)
C13	0.0316 (8)	0.0241 (9)	0.0314 (9)	−0.0045 (7)	−0.0045 (7)	−0.0022 (7)
C14	0.0427 (11)	0.0218 (8)	0.0389 (11)	−0.0087 (8)	0.0009 (8)	0.0010 (8)
C15	0.0386 (10)	0.0253 (9)	0.0348 (10)	−0.0102 (8)	0.0036 (8)	0.0030 (8)
C16	0.0269 (8)	0.0235 (8)	0.0273 (9)	−0.0033 (7)	−0.0013 (7)	0.0025 (7)
C17	0.0331 (9)	0.0310 (9)	0.0263 (9)	−0.0042 (8)	−0.0006 (7)	−0.0004 (8)
C18	0.0362 (11)	0.0439 (12)	0.0389 (11)	0.0035 (9)	0.0041 (9)	−0.0063 (9)
C19	0.0619 (14)	0.0501 (13)	0.0286 (11)	−0.0001 (12)	0.0011 (10)	0.0018 (9)
C20	0.0443 (11)	0.0285 (10)	0.0382 (10)	−0.0051 (8)	−0.0013 (9)	−0.0066 (8)

### Geometric parameters (Å, º)

**Table:**

O1—C1	1.209 (2)	C12—H12B	0.9900
O2—C1	1.326 (2)	C12—C13	1.533 (2)
O2—C11	1.4630 (19)	C13—H13	1.0000
N1—C2	1.320 (2)	C13—C14	1.530 (3)
N1—C10	1.363 (2)	C13—C20	1.525 (3)
C1—C2	1.504 (2)	C14—H14A	0.9900
C2—C3	1.411 (2)	C14—H14B	0.9900
C3—H3	0.9500	C14—C15	1.524 (3)
C3—C4	1.367 (3)	C15—H15A	0.9900
C4—H4	0.9500	C15—H15B	0.9900
C4—C5	1.410 (3)	C15—C16	1.537 (2)
C5—C6	1.419 (3)	C16—H16	1.0000
C5—C10	1.425 (2)	C16—C17	1.542 (3)
C6—H6	0.9500	C17—H17	1.0000
C6—C7	1.363 (3)	C17—C18	1.524 (3)
C7—H7	0.9500	C17—C19	1.531 (3)
C7—C8	1.413 (3)	C18—H18A	0.9800
C8—H8	0.9500	C18—H18B	0.9800
C8—C9	1.365 (3)	C18—H18C	0.9800
C9—H9	0.9500	C19—H19A	0.9800
C9—C10	1.420 (2)	C19—H19B	0.9800
C11—H11	1.0000	C19—H19C	0.9800
C11—C12	1.520 (2)	C20—H20A	0.9800
C11—C16	1.527 (2)	C20—H20B	0.9800
C12—H12A	0.9900	C20—H20C	0.9800
			
C1—O2—C11	117.76 (13)	C14—C13—H13	108.1
C2—N1—C10	117.66 (15)	C20—C13—C12	111.29 (16)
O1—C1—O2	125.27 (16)	C20—C13—H13	108.1
O1—C1—C2	122.85 (16)	C20—C13—C14	111.39 (16)
O2—C1—C2	111.88 (14)	C13—C14—H14A	109.2
N1—C2—C1	117.11 (15)	C13—C14—H14B	109.2
N1—C2—C3	124.13 (16)	H14A—C14—H14B	107.9
C3—C2—C1	118.72 (15)	C15—C14—C13	112.25 (16)
C2—C3—H3	120.7	C15—C14—H14A	109.2
C4—C3—C2	118.59 (16)	C15—C14—H14B	109.2
C4—C3—H3	120.7	C14—C15—H15A	109.4
C3—C4—H4	120.2	C14—C15—H15B	109.4
C3—C4—C5	119.69 (16)	C14—C15—C16	110.97 (16)
C5—C4—H4	120.2	H15A—C15—H15B	108.0
C4—C5—C6	123.76 (17)	C16—C15—H15A	109.4
C4—C5—C10	117.45 (16)	C16—C15—H15B	109.4
C6—C5—C10	118.79 (17)	C11—C16—C15	106.80 (14)
C5—C6—H6	119.7	C11—C16—H16	107.0
C7—C6—C5	120.70 (18)	C11—C16—C17	113.43 (15)
C7—C6—H6	119.7	C15—C16—H16	107.0
C6—C7—H7	119.9	C15—C16—C17	115.11 (15)
C6—C7—C8	120.29 (18)	C17—C16—H16	107.0
C8—C7—H7	119.9	C16—C17—H17	107.2
C7—C8—H8	119.5	C18—C17—C16	113.14 (16)
C9—C8—C7	120.93 (18)	C18—C17—H17	107.2
C9—C8—H8	119.5	C18—C17—C19	110.80 (18)
C8—C9—H9	120.0	C19—C17—C16	111.05 (17)
C8—C9—C10	120.01 (18)	C19—C17—H17	107.2
C10—C9—H9	120.0	C17—C18—H18A	109.5
N1—C10—C5	122.45 (15)	C17—C18—H18B	109.5
N1—C10—C9	118.30 (16)	C17—C18—H18C	109.5
C9—C10—C5	119.25 (16)	H18A—C18—H18B	109.5
O2—C11—H11	109.3	H18A—C18—H18C	109.5
O2—C11—C12	109.78 (14)	H18B—C18—H18C	109.5
O2—C11—C16	107.05 (14)	C17—C19—H19A	109.5
C12—C11—H11	109.3	C17—C19—H19B	109.5
C12—C11—C16	111.93 (14)	C17—C19—H19C	109.5
C16—C11—H11	109.3	H19A—C19—H19B	109.5
C11—C12—H12A	109.5	H19A—C19—H19C	109.5
C11—C12—H12B	109.5	H19B—C19—H19C	109.5
C11—C12—C13	110.90 (15)	C13—C20—H20A	109.5
H12A—C12—H12B	108.0	C13—C20—H20B	109.5
C13—C12—H12A	109.5	C13—C20—H20C	109.5
C13—C12—H12B	109.5	H20A—C20—H20B	109.5
C12—C13—H13	108.1	H20A—C20—H20C	109.5
C14—C13—C12	109.86 (15)	H20B—C20—H20C	109.5
			
O1—C1—C2—N1	−157.29 (18)	C7—C8—C9—C10	−0.4 (3)
O1—C1—C2—C3	20.5 (3)	C8—C9—C10—N1	−177.92 (16)
O2—C1—C2—N1	22.5 (2)	C8—C9—C10—C5	1.7 (3)
O2—C1—C2—C3	−159.75 (16)	C10—N1—C2—C1	178.36 (15)
O2—C11—C12—C13	−178.14 (13)	C10—N1—C2—C3	0.7 (2)
O2—C11—C16—C15	−179.03 (13)	C10—C5—C6—C7	0.3 (3)
O2—C11—C16—C17	−51.15 (19)	C11—O2—C1—O1	9.8 (3)
N1—C2—C3—C4	−1.9 (3)	C11—O2—C1—C2	−169.94 (14)
C1—O2—C11—C12	−95.24 (17)	C11—C12—C13—C14	53.7 (2)
C1—O2—C11—C16	143.06 (15)	C11—C12—C13—C20	177.55 (16)
C1—C2—C3—C4	−179.55 (17)	C11—C16—C17—C18	−59.7 (2)
C2—N1—C10—C5	1.1 (2)	C11—C16—C17—C19	174.94 (17)
C2—N1—C10—C9	−179.21 (16)	C12—C11—C16—C15	60.63 (18)
C2—C3—C4—C5	1.3 (3)	C12—C11—C16—C17	−171.49 (14)
C3—C4—C5—C6	−179.24 (18)	C12—C13—C14—C15	−53.7 (2)
C3—C4—C5—C10	0.4 (3)	C13—C14—C15—C16	57.9 (2)
C4—C5—C6—C7	179.92 (19)	C14—C15—C16—C11	−59.2 (2)
C4—C5—C10—N1	−1.7 (3)	C14—C15—C16—C17	173.92 (16)
C4—C5—C10—C9	178.65 (17)	C15—C16—C17—C18	63.7 (2)
C5—C6—C7—C8	1.1 (3)	C15—C16—C17—C19	−61.6 (2)
C6—C5—C10—N1	177.98 (16)	C16—C11—C12—C13	−59.40 (19)
C6—C5—C10—C9	−1.7 (2)	C20—C13—C14—C15	−177.47 (17)
C6—C7—C8—C9	−1.1 (3)		

### Hydrogen-bond geometry (Å, º)

**Table:**

*D*—H···*A*	*D*—H	H···*A*	*D*···*A*	*D*—H···*A*
C7—H7···N1^i^	0.95	2.56	3.509 (2)	174

Symmetry code: (i) −*x*+1, *y*−1/2, −*z*+1/2.
